# Therapeutic targets and molecular mechanism of calycosin for the treatment of cerebral ischemia/reperfusion injury

**DOI:** 10.18632/aging.203219

**Published:** 2021-06-27

**Authors:** Songzuo Yu, Ka Wu, Yujia Liang, Haitao Zhang, Chao Guo, Bin Yang

**Affiliations:** 1 Department of Neurosurgery, Guigang City People’s Hospital, The Eighth Affiliated Hospital of Guangxi Medical University, Guigang, Guangxi, PR China; 2 Department of Pharmacy, The Second People’s Hospital of Nanning City, The Third Affiliated Hospital of Guangxi Medical University, Nanning, PR China; 3 College of Pharmacy, Guangxi Medical University, Nanning, PR China; 4 Department of Neurosurgery Area 1, Guigang City People’s Hospital, The Eighth Affiliated Hospital of Guangxi Medical University, Guigang, Guangxi, PR China; 5 Department of Pharmacy, Guigang City People’s Hospital, The Eighth Affiliated Hospital of Guangxi Medical University, Guigang, Guangxi, PR China

**Keywords:** calycosin, cerebral ischemia/reperfusion injury, network pharmacology, biotargets, bioinformatics

## Abstract

This study was designed to understand the pivotal anti-cerebral ischemia/reperfusion injury (CIRI) targets and pathways of calycosin through network pharmacology and molecular docking analyses. In this study, bioinformatics tools were employed to characterize and identify the pharmacological functions and mechanisms of calycosin for CIRI management. The network pharmacology data identified potential, merged CIRI-associated targets of calycosin including tumor protein p53 (TP53), protein kinase B (AKT1), vascular endothelial growth factor A (VEGFA), interleukin 6, tumor necrosis factor (TNF), and mitogen-activated protein kinase 1 (MAPK1). Molecular docking analysis indicated the binding efficacy of calycosin with three of the targets, namely TP53, AKT1, and VEGFA. The biological processes of calycosin for the treatment of CIRI are mainly involved in the improvement of endothelial cell proliferation and growth, inflammatory development, and cellular metabolism. In addition, the anti-CIRI actions of calycosin were primarily through suppression of the toll-like receptor, PI3K-AKT, TNF, MAPK, and VEGF signaling pathways. Taken together, the current bioinformatic findings revealed pivotal targets, biological functions, and pharmacological mechanisms of calycosin for the treatment of CIRI. In conclusion, calycosin, a functional phytoestrogen, can be potentially used for the treatment of CIRI in future clinical trials.

## INTRODUCTION

Cerebral ischemia/reperfusion injury (CIRI), a type of brain injury, can cause severe damage to encephalic cells and impair their function [1]. Some common pathological manifestations detected in patients with CIRI include hydrocephalus, intracephalic necrosis, and cellular inflammatory infiltration [2]. The histopathological changes in CIRI may be short term or extended based on the hypoxemic degree of hypoxia in brain tissue. Any oxygen-deficient condition will be detrimental and lethal for the tissue as the brain is highly sensitive to hypoxia [3]. Globally, the geriatric population has been on the rise in recent decades, and the incidence of CIRI in elderly people has increased significantly resulting in a high rate of cerebrovascular disorders [4]. Epidemiological data show that cases of CIRI are mounting in hospitals, especially in less developed countries, where socioeconomic factors are poor [5]. In China, the number of cases of CIRI is higher than that in other countries and is characterized by a large economic burden [6]. Current drug therapies for CIRI management include free radical scavengers (such as edaravone), excitatory amino acid antagonists (such as coumarin), calcium channel blockers (such as nimodipine), and anti-inflammatory agents (such as lovastatin) [7]. However, the undesired effects of these pharmacotherapies commonly encountered during clinical treatment of CIRI patients are a big challenge. Natural anti-CIRI molecules can be good alternative treatment strategies to circumvent these complications and should be screened and identified accordingly. Historically, many traditional Chinese medicines have commonly been used for prophylaxis and treatment of endemic diseases, such as thyroncus and malaria [8]. Calycosin, a pharmacologically bioactive compound, has potent anti-inflammatory and antioxidant properties [9]. It has been reported that calycosin may exert potent anti-tumor activity against colorectal cancer [10]. Interestingly, the neuroprotective benefits of calycosin have been validated both *in vivo* and *in vitro* [11]. Guo et al. (2012) have established the role of calycosin as an anti-CIRI molecule in rats through induction of antioxidant effects [12]. However, the mechanism of calycosin action for treatment of CIRI remains unclear. An attractive methodology using network pharmacology and molecular docking has been applied to the discovery of therapeutic bio-targets and action pathways of bioactive compounds [13–14]. Our previous bioinformatics findings illustrate potential roles of some bioactive agents in the treatment of diseases, including calycosin against meningitis, vitamin C against COVID-19, curcumol against interstitial cystitis, and plumbagin against liver cancer [15–18].

**Figure 1:**
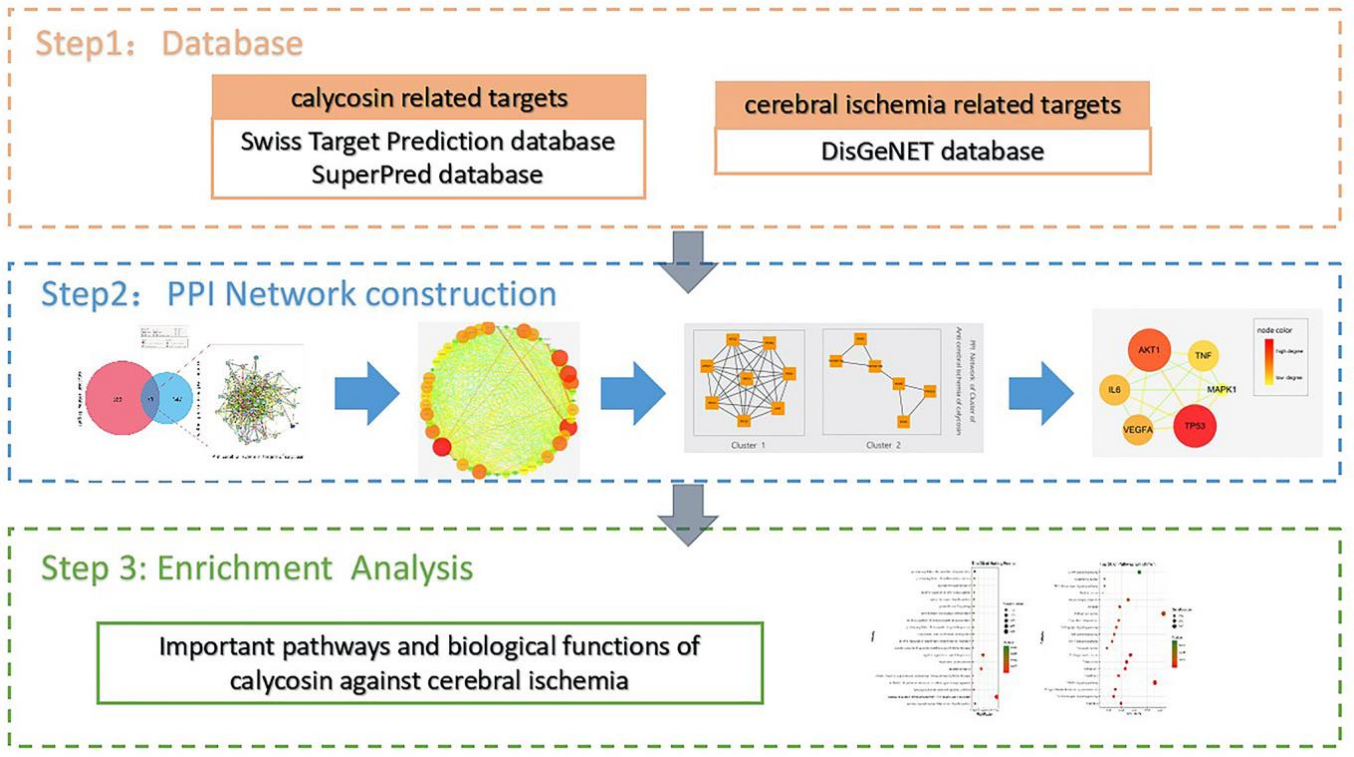
Flowchart of the current bioinformatics tools used for this study to reveal the pivotal targets and molecular mechanisms underlying the anti-CIRI action of calycosin through network pharmacology and molecular docking methods.

In the present study, we used bioinformatics tools including network pharmacology and molecular docking to uncover the pharmacological functions and therapeutic mechanisms of calycosin as an anti-CIRI agent. A flow chart was created to summarize the main findings of calycosin action for the treatment of CIRI, obtained via bioinformatics analysis (Figure 1).

## MATERIALS AND METHODS

### Harvesting the genes of calycosin-anti-CIRI target

All calycosin-putative genes were identified through a series of traditional Chinese medicine systems pharmacology (TCMSP), Swiss Target Prediction, and SuperPred databases. Meanwhile, other CIRI-associated pathogenic genes were collected using the DisGeNET and Genecards databases. Calycosin and CIRI-related genes were merged using the UniProt database. After re-determination using the Funrich software, the calycosin-anti-CIRI targets were screened and specified as described in previous reports [19–20].

### Ascertaining the pivotal targets of calycosin-anti-CIRI

All merged genes of calycosin-anti-CIRI targets were used for re-analysis to create a target-functional network via the STRING database following an associated algorithm [21]. Using Cytoscape software, all identified genes of calycosin-anti-CIRI targets were employed to plot a network of protein-protein interactions (PPI). After testing with topological parameters in the NetworkAnalyzer setting, all pivotal targets of calycosin-anti-CIRI were identified, and these key targets were visualized in a network diagram [22].

### Analyses of key targets in biological functions and molecular mechanisms

The pharmacological functions and signaling pathways of all pivotal targets of calycosin-anti-CIRI were identified and uncovered using the Database for Annotation, Visualization, and Integrated Discovery (DAVID) database. Subsequently, pivotal targets were determined using Cytoscape software to create a network diagram of anti-CIRI targets of calycosin. After determining the log P value using the OmicShare tool, the advanced bubble diagrams used for biological processes and Kyoto Encyclopedia of Genes and Genomes (KEGG)-based molecular pathways were detailed and visualized, as reported previously [23–24].

### Molecular docking of calycosin with anti-CIRI targets

In brief, pivotal targets including tumor protein p53 (TP53), protein kinase B (AKT1), and vascular endothelial growth factor A (VEGFA) were screened and used for calycosin-associated molecular docking. The structure of these target proteins was retrieved from protein-data-bank (PDB) database with PDB IDs 2MWO, 3O96, and 5T89, respectively. Calycosin was docked to these proteins at their respective binding sites where their ligands are bound. The three-dimensional structure of calycosin prior to docking was demonstrated using ChemBio3D Draw setting of Chem Bio Office 2010 software. The software AutoDock Vina was used for all dockings and the molecular docked structures of calycosin with the target proteins of TP53, AKT1, and VEGFA were viewed using MGLTools in Autodock Vina software. The accuracy of the docking parameters was determined and identified using the root mean square deviation (RMSD) of the ligand molecules. The setting with an RMSD <4 Å is denoted as the threshold to conform to ligand molecules [25–26].

## RESULTS

### Harvested targets of calycosin and CIRI

**Figure 2:**
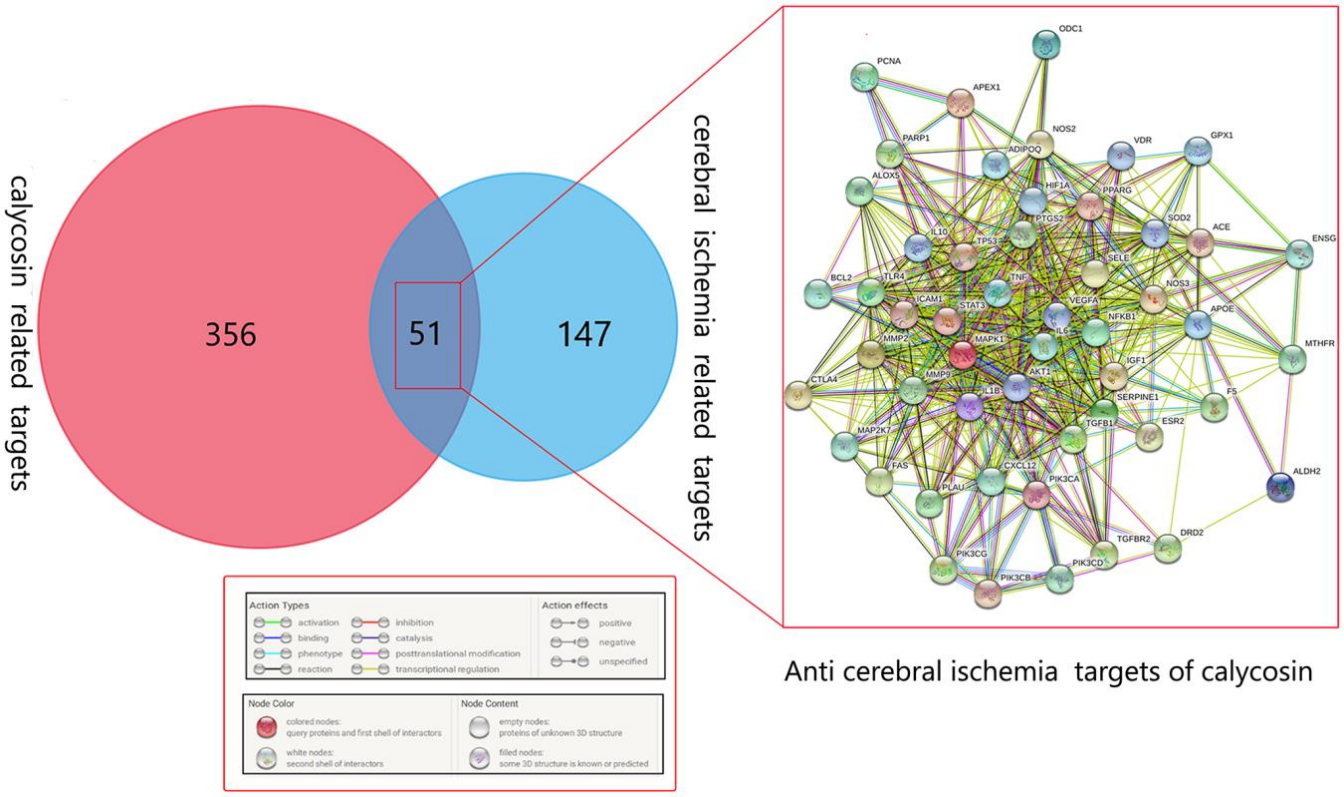
**Venn diagram showing all the common, merged targets of calycosin and CIRI.** All merged targets are interrelated in the network diagram.

After analyses of databases, 407 anti-diseased targets of calycosin and 198 diseased targets of CIRI were identified. In addition, a total of 51 merged targets of calycosin and CIRI are shown in a Venn diagram. The correlation network connected with these merged targets is shown in Figure 2.

### Cluster analysis findings

**Figure 3:**
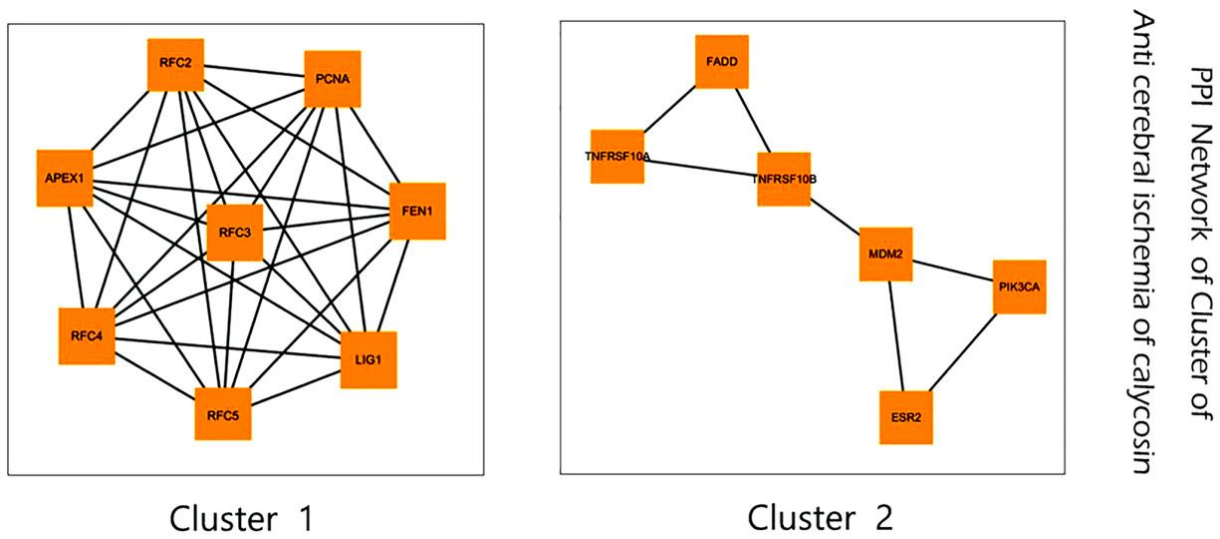
Subnetwork clusters of identified targets for calycosin against CIRI obtained using MCODE algorithm.

In the following analysis, the sub-network clusters obtained by the MCODE algorithm in Cytoscape software are shown in Figure 3. The predictive targets of RFC2, PFC3, RFC4, RFC5, FEN1, LIG1, PCNA, and APEX1 in calycosin against CIRI were clustered as a category. Other FADD, TNFRSF10A, TNFRSF10B, MDM2, PIK3CA, and ESR2 targets in calycosin against CIRI were grouped as another category.

### Pivotal target findings

**Figure 4:**
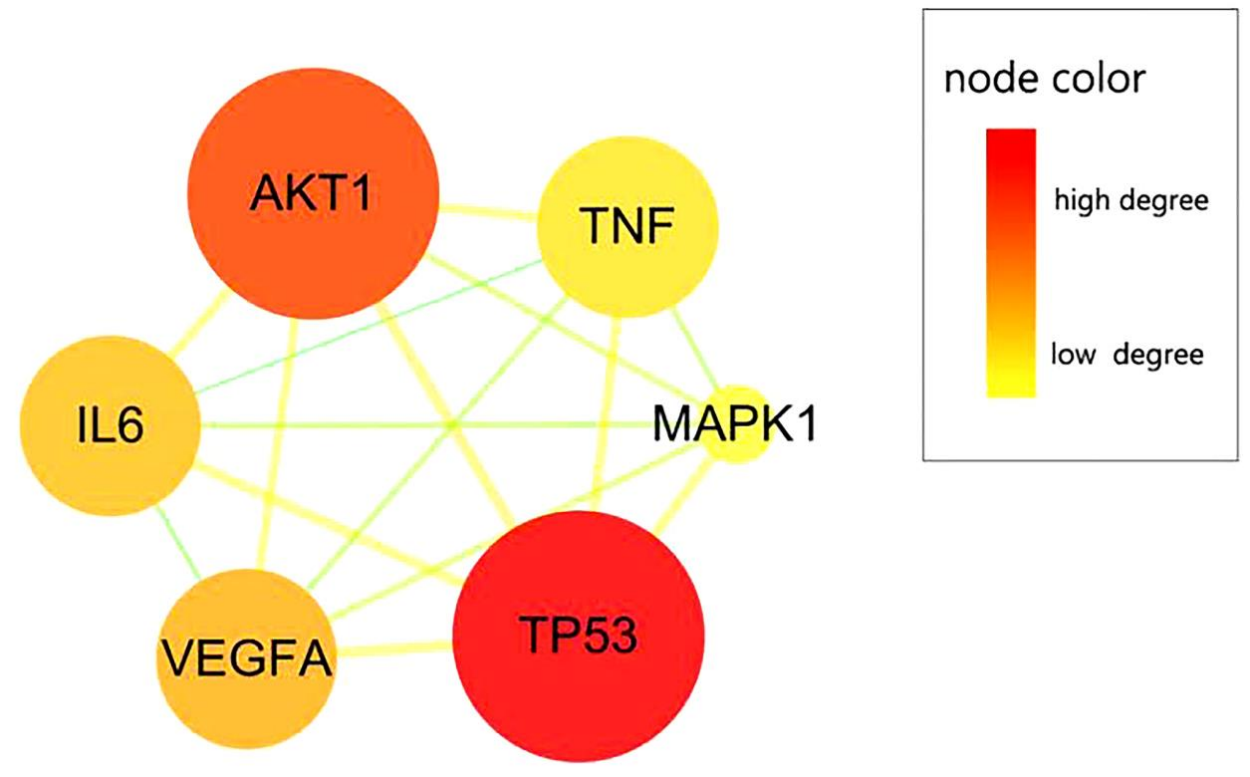
**CIRI-related pivotal targets of calycosin.** Six pivotal targets were screened and identified from merged targets, namely TP53, AKT1, VEGFA, IL6, TNF, and MAPK1.

Based on the topological data and degree value to screen pivotal targets, the parameters of mean and large degrees of freedom in calycosin against anti-IRI target proteins were standardized. As a result, six core CIRI-associated targets of calycosin were screened and identified, including TP53, AKT1, VEGFA, interleukin 6 (IL6), tumor necrosis factor (TNF), and mitogen-activated protein kinase 1 (MAPK1) (Figure 4).

### Biological functions and anti-CIRI pathways

**Figure 5:**
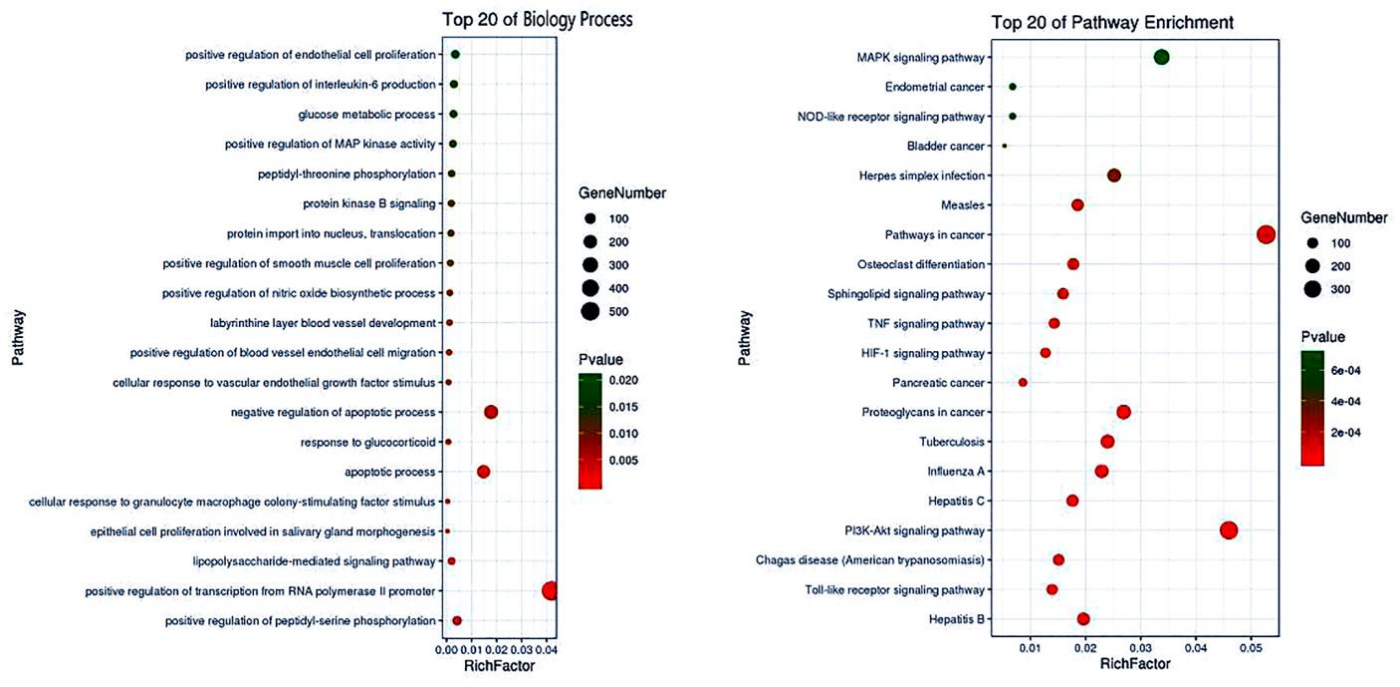
**Top 20 pharmacological processes and molecular pathways of calycosin for CIRI management.** Based on enrichment analysis, both pharmacological processes and molecular pathways were revealed to understand the mechanism of action of calycosin for the treatment of CIRI.

We revealed the pharmacological mechanisms of calycosin for management of CIRI using enrichment analysis with pivotal targets. An advanced bubble chart showed top 20 biological processes of calycosin that can possibly be involved for treatment of CIRI, including its metabolic and apoptotic functions. The detailed mechanisms of calycocin action were determined from the enrichment analysis findings that revealed top 20 anti-CIRI pathways including modulation of cell proliferation and inflammation, and improvement of intracellular microenvironment (Figure 5). More data with a total of 29 biological processes and 78 molecular pathways of calycosin for CIRI management are provided in Supplementary Table 1 and Supplementary Table 2 respectively.

### Molecular docking findings

**Figure 6:**
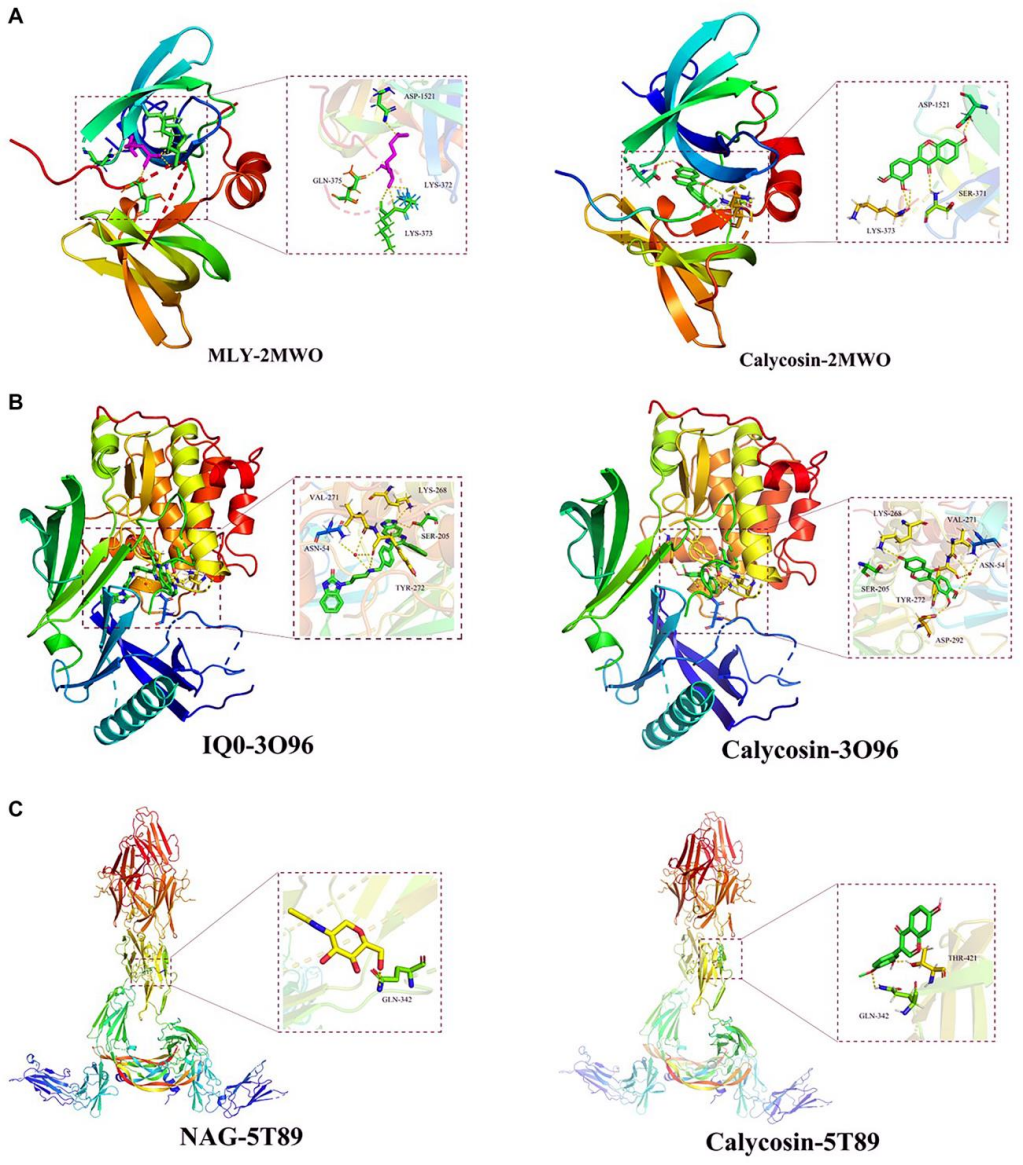
**Docking poses of calycosin on three identified targets.** By using molecular docking analysis, the data demonstrated that effective binding capacities of calycosin with CIRI were identified in (**A**) TP53 (2MWO), (**B**) AKT1 (3O96), and (**C**) VEGFA (5T89) targets.

The RMSD value of MLY, the original bound ligand in the TP53 protein (2MWO) was 2.675 Å. The ligand was bound at the target site through hydrogen bonding with the amino acid residues of the protein including ASP-1521 (2.7 Å), LYS-372 (2.6 Å), GLN-375 (1.8 Å), and LYS-373 (2.3 Å). In the calycosin-docked protein, calycosin was found to make hydrogen bonds with amino acid residues of ASP-1521 (2.5 Å), LYS-373 (2.4 Å), and SER-371 (2.5 Å) (Figure 6A). In AKT1 (3O96), the RMSD value of IQ0, the original ligand, was 1.024 Å. The hydrogen bonding of the ligand with the binding site amino acid residues of the protein included VAL-271 (3.2Å), ASN-54 (2.9 Å), LYS-268 (2.6 Å), SER-205 (2.5 Å), and TYR-272 (3.2 Å).

Calycosin formed hydrogen bonds with the amino acid residues of VAL-271 (3.2 Å), ASN-54 (2.9 Å), LYS-268 (2.4 Å), SER-205 (2.4 Å), TYR-272 (3.3 Å), and ASP-292 (2.3 Å) (Figure 6B). In the VEGFA protein (5T89), the RMSD value of the original ligand, NAG, was 3.130 Å. The amino acid residue of the protein involved in hydrogen bonding with the ligand was GLN-342 (3.2 Å). Calycosin was involved in docking interaction with the amino acid residues, GLN-342 (2.6 Å) and THR-421 (2.0 Å) after being docked to VEGFA protein (Figure 6C).

## DISCUSSION

Our current findings via bioinformatics analyses using network pharmacology and molecular docking revealed pivotal targets, biological functions, and molecular pathways of calycosin involved in CIRI management. We identified a total of six pivotal CIRI-associated targets of calycosin, including TP53, AKT1, VEGFA, IL6, TNF, and MAPK1. Moreover, molecular docking analysis demonstrated efficient binding of calycosin with thee of the targets, namely TP53, AKT1, and VEGFA. These findings strongly implicate an anti-CIRI action of calycosin. TP53, a well-reported anti-oncogene, can suppress intracellular DNA injury or genomic aberrations that are responsible for cell cycle arrest and cell growth [27]. When mutated, the variant TP53 in diseased tissues may induce tumorigenesis, causing human tumor growth [28]. Increasing evidence shows a neuroprotective effect of activated TP53 against spiral ganglion neuron injury in mice through regulation of the Wnt signaling pathway [29]. AKT1, a protein kinase, plays an essential role in controlling cell survival and apoptosis [30]. It has been experimentally found that activation of AKT1 signaling may inhibit neurodegeneration in amyloid β-deposited brains in rats [31]. VEGFA, a vascular endothelial growth factor, is involved in regulating vascular endothelial cell growth, vascular permeability, and angiogenesis [32]. Some data indicate that VEGFA overexpression in Müller cells, the principal glial cells, may promote retinal dysfunction [33]. Although current evidence establishes a role of these targets in neuroprotection, reports indicating roles of their genes in anti-CIRI actions are limited. Therefore, it is reasoned that TP53, AKT1, and VEGFA genes may function as effective neuroprotection agents against CIRI. Enrichment analysis revealed that the biological processes of calycosin for CIRI management are involved in the amelioration of endothelial cell proliferation and growth, inflammatory development, and cellular metabolism. These functions might be primarily responsible for the pharmacological action of calycosin in the treatment of CIRI. Mechanically, the anti-CIRI action of calycosin is via inhibition of the toll-like receptor, PI3K-AKT, TNF, MAPK, and VEGF signaling pathways. These data indicate that calycosin might contribute to the suppression of neuroinflammation, neural lesion/necrosis, and vascular degeneration. Despite absence of any experimental validation, our current bioinformatics findings indicate that calycosin may be a promising candidate for the treatment of CIRI.

## CONCLUSION

In conclusion, the pivotal targets, biological functions, and molecular mechanisms of calycosin related to the treatment of CIRI are revealed through bioinformatics tools using network pharmacology and molecular docking. Furthermore, pharmacological targets, including TP53, AKT1, and VEGFA, have been identified before any experimental validation.

## Supplementary Materials

Supplementary Tables
