# Supplementary material for: Therapeutic targets and molecular mechanism of calycosin for the treatment of cerebral ischemia/reperfusion injury
Source: Aging (Albany NY). 2021 Jun 27;13(12):16804–15. doi: 10.18632/aging.203219 (PMC8266369; doi:10.18632/aging.203219)
Supplement: Supplementary Tables [file aging-13-203219-s001.pdf]

SUPPLEMENTARY TABLES

Supplementary Table 1.

Term	Pop Hits	Pop Total	*P* Value
positive regulation of peptidyl-serine phosphorylation	58	13824	1.39E-06
positive regulation of transcription from RNA polymerase II promoter	580	13824	4.30E-05
lipopolysaccharide-mediated signaling pathway	28	13824	5.90E-05
epithelial cell proliferation involved in salivary gland morphogenesis	5	13824	0.002169
cellular response to granulocyte macrophage colony-stimulating factor stimulus	6	13824	0.002602
apoptotic process	204	13824	0.003126
response to glucocorticoid	10	13824	0.004333
negative regulation of apoptotic process	246	13824	0.004513
cellular response to vascular endothelial growth factor stimulus	11	13824	0.004766
positive regulation of blood vessel endothelial cell migration	14	13824	0.006062
labyrinthine layer blood vessel development	16	13824	0.006926
positive regulation of nitric oxide biosynthetic process	18	13824	0.007789
positive regulation of smooth muscle cell proliferation	21	13824	0.009082
protein import into nucleus, translocation	24	13824	0.010373
protein kinase B signaling	26	13824	0.011234
peptidyl-threonine phosphorylation	28	13824	0.012094
positive regulation of MAP kinase activity	35	13824	0.015098
glucose metabolic process	38	13824	0.016383
positive regulation of interleukin-6 production	40	13824	0.017239
positive regulation of endothelial cell proliferation	48	13824	0.020657
cellular response to hypoxia	50	13824	0.02151
response to exogenous dsRNA	51	13824	0.021936
humoral immune response	56	13824	0.024065
positive regulation of protein phosphorylation	59	13824	0.02534
positive regulation of sequence-specific DNA binding transcription factor activity	65	13824	0.027887
glucose homeostasis	86	13824	0.036757
peptidyl-serine phosphorylation	112	13824	0.047646
defense response to virus	125	13824	0.053051
positive regulation of apoptotic process	133	13824	0.056365

Supplementary Table 2.

Term	Pop Hits	Pop Total	*P* Value
Hepatitis B	148	7550	1.60E-08
Toll-like receptor signaling pathway	105	7550	5.19E-07
Chagas disease (American trypanosomiasis)	114	7550	7.23E-07
PI3K-Akt signaling pathway	347	7550	1.15E-06
Hepatitis C	133	7550	1.34E-06
Influenza A	173	7550	3.85E-06
Tuberculosis	181	7550	4.62E-06
Proteoglycans in cancer	203	7550	7.30E-06
Pancreatic cancer	65	7550	1.20E-05
HIF-1 signaling pathway	96	7550	3.88E-05
TNF signaling pathway	108	7550	5.52E-05
Sphingolipid signaling pathway	120	7550	7.56E-05
Pathways in cancer	398	7550	1.05E-04
Osteoclast differentiation	134	7550	1.05E-04
Measles	140	7550	1.20E-04
Herpes simplex infection	190	7550	2.97E-04
Bladder cancer	40	7550	4.05E-04
NOD-like receptor signaling pathway	51	7550	6.60E-04
Endometrial cancer	51	7550	6.60E-04
MAPK signaling pathway	255	7550	7.06E-04
Non-small cell lung cancer	56	7550	7.95E-04
HTLV-I infection	267	7550	8.08E-04
mTOR signaling pathway	59	7550	8.83E-04
VEGF signaling pathway	59	7550	8.83E-04
Apoptosis	62	7550	9.74E-04
Central carbon metabolism in cancer	62	7550	9.74E-04
Glioma	65	7550	0.001071
Renal cell carcinoma	66	7550	0.001104
Colorectal cancer	66	7550	0.001104
Fc epsilon RI signaling pathway	67	7550	0.001137
Melanoma	72	7550	0.001312
Chronic myeloid leukemia	73	7550	0.001349
Pertussis	77	7550	0.0015
Prostate cancer	86	7550	0.001867
Rheumatoid arthritis	95	7550	0.002274
T cell receptor signaling pathway	105	7550	0.002771
Insulin resistance	111	7550	0.003092
Thyroid hormone signaling pathway	113	7550	0.003202
Toxoplasmosis	113	7550	0.003202
Natural killer cell mediated cytotoxicity	117	7550	0.003429
Neurotrophin signaling pathway	125	7550	0.003905
FoxO signaling pathway	132	7550	0.004346
Jak-STAT signaling pathway	151	7550	0.005654
Non-alcoholic fatty liver disease (NAFLD)	160	7550	0.00633
Focal adhesion	208	7550	0.010532
Rap1 signaling pathway	214	7550	0.011126

Cytokine-cytokine receptor interaction	218	7550	0.01153
Ras signaling pathway	235	7550	0.013322
Thyroid cancer	31	7550	0.024392
Prion diseases	32	7550	0.025171
African trypanosomiasis	37	7550	0.029056
Graft-versus-host disease	42	7550	0.032927
Type II diabetes mellitus	47	7550	0.036786
Malaria	53	7550	0.0414
Amyotrophic lateral sclerosis (ALS)	54	7550	0.042168
Acute myeloid leukemia	56	7550	0.0437
Legionellosis	57	7550	0.044466
Cytosolic DNA-sensing pathway	61	7550	0.047524
B cell receptor signaling pathway	70	7550	0.054373
Inflammatory bowel disease (IBD)	70	7550	0.054373
Leishmaniasis	71	7550	0.055132
Adipocytokine signaling pathway	71	7550	0.055132
Prolactin signaling pathway	74	7550	0.057404
RIG-I-like receptor signaling pathway	76	7550	0.058917
Hypertrophic cardiomyopathy (HCM)	80	7550	0.061936
TGF-beta signaling pathway	83	7550	0.064194
Salmonella infection	83	7550	0.064194
Fc gamma R-mediated phagocytosis	85	7550	0.065698
ErbB signaling pathway	86	7550	0.066449
Small cell lung cancer	87	7550	0.067199
Progesterone-mediated oocyte maturation	88	7550	0.067949
Hematopoietic cell lineage	92	7550	0.070944
Estrogen signaling pathway	98	7550	0.075421
Choline metabolism in cancer	99	7550	0.076165
Cholinergic synapse	110	7550	0.084321
Amoebiasis	112	7550	0.085798
Epstein-Barr virus infection	118	7550	0.090215
Platelet activation	127	7550	0.096808
